# Serum levels of SIRT3 and other inflammatory factors are associated with clinical outcomes and prognosis in severe community-acquired pneumonia in adults: A prospective study

**DOI:** 10.1097/MD.0000000000026721

**Published:** 2021-08-13

**Authors:** Wei Zhu, Ping Chen, Liangzi Hu, Li Deng

**Affiliations:** a Department of Critical Care, Tianyou Hospital Affiliated to Wuhan University of Science and Technology, Wuhan, China.; b Department of General practice, Tianyou Hospital Affiliated to Wuhan University of Science and Technology, Wuhan, China.; c Department of Pharmacy, Tianyou Hospital Affiliated to Wuhan University of Science and Technology, Wuhan, China.

**Keywords:** inflammation, prognosis, severe community-acquired pneumonia, SIRT3

## Abstract

The aim of this study was to investigate clinical significance of SIRT3 in severe community-acquired pneumonia (CAP) patients.

This prospective observational research enrolled a total of 114 severe CAP patients who went to our hospital during January 2018 to December 2019. Serum SIRT3 and IL-1β, IL-6, and tumor necrosis factor (TNF)-α levels were determined using the enzyme-linked immunosorbent assay (ELISA) method. Demographic data, including age, sex, and body mass index (BMI), as well as clinical symptoms, SOFA and SMART-COP scores were collected. The routine blood test was conducted for all patients and white blood cell (WBC) amount, as well as serum levels of C-reactive protein (CRP), D-Dimer, and procalcitonin (PCT).

Among all patients, 55 cases died during the study period. The serum levels of CRP, PCT, IL-1β, and IL-6, as well as SOFA and SMART-COP scores were markedly higher in deceased patients than in the survival patients. The expression of SIRT3 was significantly decreased in severe CAP patients compared with the healthy, especially in the deceased patients. SIRT3 levels were negatively correlated with levels of CRP, PCT, IL-1β, and IL-6. Patients with SIRT3 low expression showed remarkably higher expression of CRP, PCT, IL-1β, and IL-6, as well as high SMART-COP scores, higher 1-month mortality rate, and shorter survival. Only SIRT3 and IL-1β were independent risk factors for 1-month mortality in severe CAP patients.

Lower serum SIRT3 level predicts poor clinical outcomes and prognosis in severe CAP patients.

## 1 Introduction

Community-acquired pneumonia (CAP), a kind of infectious pulmonary parenchymal inflammation (including alveolar wall, or pulmonary interstitium in a broad sense) outside the hospital, is a common respiratory disease with high incidence and mortality rates.^[1,2]^ It was reported that CAP accounts for more than 3 million deaths annually worldwide.^[3,4]^ As CAP, especially severe CAP, often has high mortality rate, biomarkers for diagnosis and prognosis are of great significance.^[5–7]^

Inflammation is deeply associated with CAP development. During CAP, inflammatory response is activated and the secretion of inflammatory factors is increased.^[8,9]^ It was reported that cytokines such as NF-κB, IL-17, TNF-α, IFN-γ, and IL-4 were correlated with clinical severity scales such as CURB65 or SOFA in CAP patients.^[10]^ And white blood cell (WBC), neutrophil, monocyte, lymphocyte ratio (NLR), platelet to lymphocyte ratio (PLR), and monocyte to lymphocyte ratio (MLR) levels were all higher in the CAP patients than the healthy individuals.^[11]^

Sirtuin 3 (SIRT3) is a member of Sirtuin family, which play important roles in many diseases. It was found SIRT3 could regulate mitochondrial fatty-acid oxidation by reversible enzyme deacetylation.^[12]^ Other studies demonstrated that SIRT3 played multiple roles in cardiovascular diseases,^[13]^ cancer,^[14]^ and neurodegenerative diseases.^[15]^ In recent years, SIRT3 is found to show anti-inflammation activity in many diseases.^[16]^ However, up to now, no study focused on clinical significance of SIRT3 in severe CAP patients.

In the present study, we performed a prospective observational research to investigate the role of SIRT3 in severe CAP patients. This study might provide clinical evidence for prognostic value of SIRT3 in CAP.

## 2 Methods and materials

### 2.1 Patients

This prospective observational research enrolled a total of 114 severe CAP patients who went to our hospital during January 2018 to December 2019. The diagnosis of CAP was according to the criteria of the American Thoracic Society (ATS) guidelines for pneumonia.^[17]^ All patients were within 18–90 years-old. The severity of CAP was defined by pneumonia severity index (PSI) score, stage IV-V as severe CAP. The main inclusion criteria were CAP patients who needed to maintain breathing by invasive mechanical ventilation through tracheal intubation; patients with septic shock and blood pressure drop and need to use antihypertensive drugs to maintain blood pressure. The secondary criteria were respiratory rate > 30/min; PaO_2_/FiO_2_≤250; patients with drowsiness, disturbance of consciousness, lethargy, coma, delusion; obvious increase of serum levels of creatinine (Cr) and blood urea nitrogen (BUN) caused by CAP; patchy or patchy infiltrations in multiple lobes with or without pleural effusion by CT or X-ray; WBC < 4.0 × 10^9^/L; PLT < 100 × 10^12^/L; temperature < 36^o^C. Patients meeting one of the main inclusion criteria or 3 or more of the secondary criteria were defined as severe CAP. The following patients were excluded: patients with cancers; patients with severe renal or liver dysfunction before diagnosis of CAP; patients with cardiovascular diseases such coronary heart disease and heart failure; and patients with pulmonary tuberculosis, lung infarction, lung disease caused by autoimmune diseases, lung infection after organ transplantation. In addition, blood samples of 114 healthy individuals who went to physical examination were collected during the same period. All patients signed the informed consent. The present study was approved by the Ethic Committee of Tianyou Hospital Affiliated to Wuhan University of Science and Technology.

### 2.2 Measurement of serum SIRT3 and inflammatory factors

Fasting cubital venous blood (5 mL) of all patients were collected within 24 hours after admission. The blood samples were collected in tubes containing EDTA and were centrifuged at 2000 g for 15 minutes. The enzyme-linked immunosorbent assay (ELISA) method was used for measurement of serum SIRT3, interleukin (IL)-1β, IL-6, and tumor necrosis factor (TNF)-α using commercially available ELISA kits (SIRT3, MBS2022533 MYBio; IL-1β DLB50 R&D Systems; IL-6 MBS175877 MYBio; TNF-α DTA00D R&D Systems).

### 2.3 Data collection

Demographic data including age, sex, and body mass index (BMI), as well as clinical symptoms, comorbidities, SOFA and SMART-COP scores, duration of ICU stay, and duration of mechanical ventilation were collected. The routine blood test was conducted for all patients and WBC amount, as well as serum levels of C-reactive protein (CRP), D-Dimer, and procalcitonin (PCT). For 1-month survival analysis, all course death for patients was considered and the survival duration was defined from the admission time to the death or the last follow-up.

### 2.4 Statistical analysis

The distribution of the data was analyzed by Kolmogorov--Smirnov method. The data distributing normally was expressed as mean ± SD. Normally distributed data were expressed by mean ± SD and non-normally distributed data were expressed by median (range). Chi-square test was used to compare the rates. Comparison between 2 groups was analyzed by Student *t* test and Mann--Whitney *U* test for normally and non-normally distributed data, respectively. Correlation between SIRT and inflammatory factors was determined using Pearman analysis. Kaplan--Meier curve was performed for survival analysis. Logistic regression analysis was conducted for 1-month mortality using binary regression analysis by a step back method. *P* < .05 was considered as statistically different. All calculations were made using SPSS 18.0 (SPSS Inc., Chicago, IL).

## 3 Results

### 3.1 Characteristics of all patients

The present study included 114 severe CAP patients, with median age 59.5 (45∼79) years, male: female 63: 51. No significant difference was found between the health and the CAP patients for age, sex, and BMI. Among all patients, 55 cases died during the study period. The serum levels of CRP, PCT, IL-1β, and IL-6, as well as SOFA and SMART-COP scores were markedly higher in deceased patients compared with the survival patients (*P* < .05, Table 1).

**Table 1:** Characteristics of all patients.

Variable	All severe CAP patients, n = 114	Survival, n = 69	Deceased, n = 45	Health, n = 114	*P* ^∗^
Mean age, yr	59.5 (45∼79)	59 (45∼79)	63 (45∼79)	60 (45∼80)	.385
Sex, male: female	63: 51	39: 30	24: 21	60: 54	.734
BMI, kg/m^2^	23.36 (19.01∼26.99)	22.95 (19.29∼26.99)	23.69 (19.01∼26.78)	23.30 (19.03∼26.98)	.454
Comorbidities, n (%)					.954
Hypertension	59 (51.75)	34 (49.28)	25 (55.56)	-	
Diabetes	28 (24.56)	16 (23.19)	12 (26.67)	-	
Duration of ICU stay, d	15.04 ± 5.66	15.60 ± 4.84	14.17 ± 6.70	-	.189
Duration of mechanical ventilation, h	215.36 ± 118.76	203.47 ± 108.78	233.60 ± 131.79	-	.187
Symptoms, n (%)					.596
Fever	56 (49.12)	36 (52.17)	20 (44.44)		
Cough	43 (37.72)	28 (40.58)	15 (33.33)		
Sputum	31 (27.19)	18 (26.09)	13 (28.89)		
Shortness of breath	24 (21.05)	13 (18.84)	11 (24.44)		
Chest pain	22 (19.30)	12 (17.39)	10 (22.22)		
SOFA	5 (1∼11)	5 (1∼8)	6 (3∼11)		.044
SMART-COP	4 (1∼9)	3 (1∼5)	6 (2∼9)		<.001
CRP, mg/L	39.50 (10.4∼100.09)	33.01 (15.59∼49.63)	62.69 (10.88∼99.28)	5.20 (1.06∼9.93)	<.001
PCT, μg/mL	32.01 (10.21∼89.43)	26.11 (10.21∼38.84)	54.60 (20.48∼89.43)	5.51 (1.19∼9.98)	<.001
D-Dimer, μg/mL	7.47 ± 3.96	7.65 ± 3.95	7.19 ± 4.00	0.55 ± 0.26	.545
WBC, 10^9^/mL	13.53 (7.06∼18.98)	13.98 (7.44∼18.88)	12.14 (7.06∼18.98)	5.51 (1.10∼9.97)	.730
IL-1β, pg/mL	46.88 ± 22.69	34.54 ± 15.29	65.80 ± 18.89	5.58 ± 2.60	<.001
IL-6, pg/mL	51.75 (11.19∼112.47)	41.17 (11.19∼64.34)	80.38 (46.41∼112.47)	5.26 (1.20∼9.94)	<.001
TNF-α, pg/mL	55.43 ± 23.26	57.07 ± 22.55	52.91 ± 24.36	4.88 ± 2.46	.353

### 3.2 Serum SIRT3 levels were decreased in severe CAP patients and were negatively correlated with inflammatory factors

**Figure 1:**
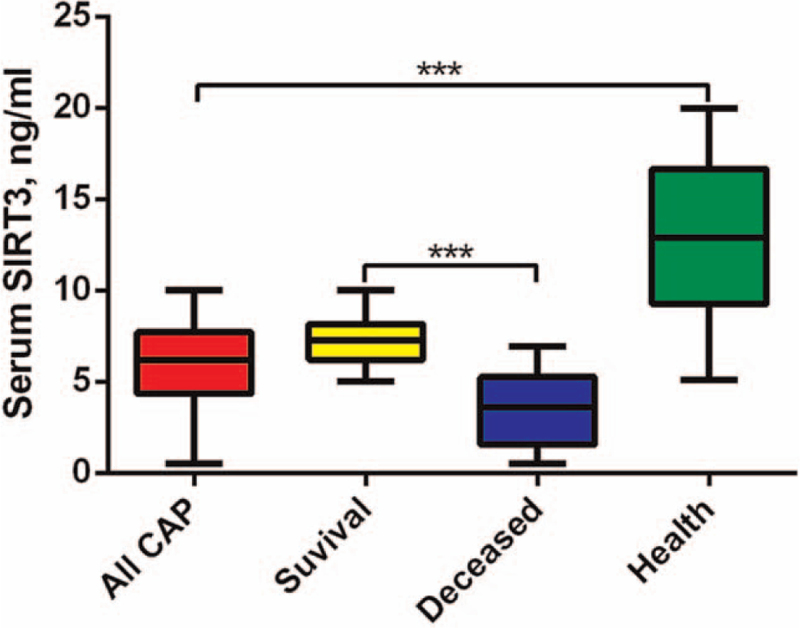
Serum levels of SIRT3 in severe CAP patients and the healthy control.

Then, we determined serum SIRT3 levels in CAP patients and the health control. As shown in Figure 1, the expression of SIRT3 was significantly decreased in severe CAP patients compared with the healthy (*P* < .05). The deceased patients showed remarkably lower SIRT3 levels compared with the survival cases (*P* < .05). Pearson analysis showed that SIRT3 levels were negatively correlated with levels of CRP, PCT, IL-1β, and IL-6 (*P* < .05, Table 2).

**Table 2:** Pearson analysis for correlation among SIRT3 and inflammatory factors.

Indices	Pearson correlation	*P*
CRP	−0.581	<.001
PCT	−0.585	<.001
IL-1β	−0.570	<.001
IL-6	−0.537	<.001
TNF-α	0.100	.291
D-Dimer	−0.082	.383

### 3.3 Serum SIRT3 levels were associated with clinical outcomes of severe CAP patients

To further investigate role of SIRT3 in severe CAP severe patients, the patients were divided into low SIRT3 group and high SIRT3 group according to the median value of serum SIRT3 (5.66 ng/mL). As summarized in Table 3, patients with SIRT3 low expression showed remarkably higher expression of CRP, PCT, IL-1β and IL-6, as well as high SMART-COP scores (*P* < .05). Besides, the SIRT3 low group also showed higher 1-month mortality rate. All these results indicated that SIRT3 was associated with clinical outcomes and prognosis of severe CAP patients.

**Table 3:** Clinical outcomes between SIRT3 low/high groups.

Variable	SIRT3 low, n = 57	SIRT3 high, n = 57	*P*
Mean age, yr	61 (45∼79)	58 (45∼79)	.401
Sex, male: female	34: 23	29: 28	.212
BMI, kg/m^2^	23.53 (19.01∼26.99)	23.31 (19.29∼26.83)	.984
Comorbidities, n (%)			.784
Hypertension	33 (57.89)	26 (45.61)	
Diabetes	15 (26.32)	13 (22.81)	
Duration of ICU stay, d	14.89 ± 6.33	15.19 ± 4.96	.780
Duration of mechanical ventilation, h	234.10 ± 121.47	196.63 ± 113.97	.092
Symptoms, n (%)			.298
Fever	33 (57.89)	23 (40.35)	
Cough	20 (35.09)	23 (40.35)	
Sputum	18 (31.58)	13 (22.81)	
Shortness of breath	11 (19.30)	13 (22.81)	
Chest pain	10 (17.54)	12 (21.05)	
SOFA	5 (1∼11)	5 (1∼8)	.171
SMART-COP	5 (1∼9)	3 (1∼7)	.001
CRP, mg/L	43.72 (10.88∼99.28)	33.01 (15.59∼93.57)	.002
PCT, μg/mL	41.51 (10.21∼89.43)	27.33 (10.74∼72.48)	<.001
D-Dimer, μg/mL	7.95 ± 4.14	6.99 ± 3.73	.194
WBC, 10^9^/mL	12.37 (7.48∼18.87)	14.15 (7.06∼18.98)	.583
IL-1β, pg/mL	57.28 ± 23.05	36.48 ± 16.96	<.001
IL-6, pg/mL	65.91 (12.03∼112.47)	41.17 (11.19∼108.09)	<.001
TNF-α, pg/mL	54.58 ± 24.95	56.28 ± 21.63	.698
1-month mortality, n (%)	41 (71.93)	4 (7.02)	<.001

### 3.4 Serum SIRT3 levels were associated with 1-month mortality of severe CAP patients

**Figure 2:**
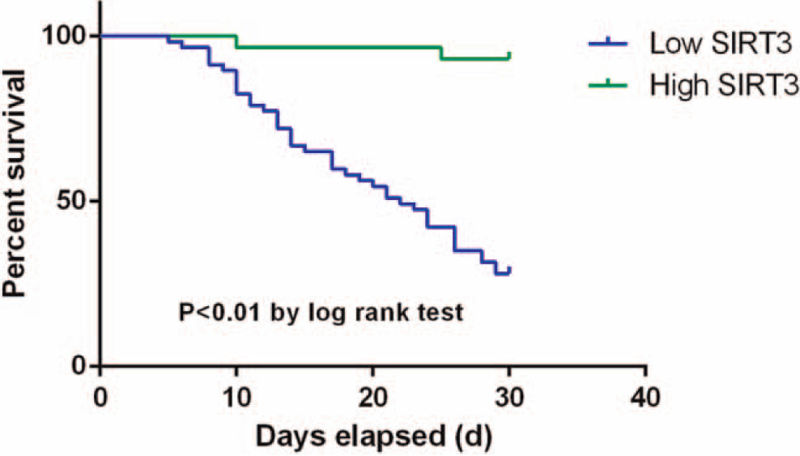
K-M curve for severe CAP patients with low/high SIRT3 expression.

Finally, survival analysis was conducted for 1-month mortality of all patients. As the univariate analysis for 1-month mortality has been already summarized in Table 1, the binary regression was performed for the factors which showed significant difference in univariate analysis. It was found patients with higher expression of SIRT3 showed significantly lower 1-month mortality rate and longer survival (Fig. 2). Logistic regression showed only SIRT3 and IL-1β were independent risk factors for 1-moth mortality in severe CAP patients (Table 4).

**Table 4:** Logistic regression for risk factors of 1-month mortality in severe CAP patients.

	Wald	Odds ratio	95% CI	*P*
SOFA	1.699	2.026	(0.700∼5.861)	.192
SMART-COP	2.930	1.952	(0.907∼4.201)	.087
SIRT3	13.317	0.207	(0.089∼0.483)	<.001
CRP	1.990	1.073	(0.972∼1.184)	.158
PCT	2.846	1.289	(0.959∼1.733)	.092
IL-1β	10.582	1.104	(1.040∼1.171)	.001
IL-6	3.308	1.261	(0.982∼1.620)	.069
TNF-α	2.563	0.928	(0.847∼1.016)	.109

## 4 Discussion

Despite the development of medical techniques, CAP is still commonly seen in clinic with a high morbidity rate. Clinical biomarkers for CAP are of great significance for diagnosis and prognosis, and novel biomarkers are always needed. In the present study, we demonstrated that serum SIRT3 levels were decreased in severe CAP patients and were associated with patients’ clinical outcomes and prognosis.

SIRT3 showed its anti-inflammation activity in many studies. It was found that SIRT3 could improve inflammation in endotoxin-induced acute lung injury.^[18]^ The activation of SIRT3 showed anti-inflammation activity in both liver and renal injury.^[19,20]^ In a recent study, Dikalova et al^[21]^ also found that the deficiency of SIRT3 might be associated with vascular dysfunction, increased vascular inflammation and oxidative stress in hypertension. The role of SIRT3 in inflammation might be partly associated with its effects on oxidative stress and energy metabolism. It has been found in cardiovascular diseases, SIRT3 protects heart from metabolic dysfunction by regulating glucose and lipid metabolism and making balance for myocardial ATP.^[22]^ In neurodegenerative disease, SIRT3 also shows its anti-oxidative stress activity.^[15]^ It is well-known that there is crosstalk between inflammation and oxidative stress, in which activated oxidative stress might induce release of pro-inflammatory factors.^[23]^ Thus, it is not surprise for SIRT3 to show its anti-inflammatory activity in multiple diseases. In this research, we found for the first time that SIRT3 was associated with prognosis of severe CAP. It was observed that SIRT3 levels were negatively correlated with levels of CRP, PCT, IL-1β, and IL-6, which play important roles in CAP development.

It is widely accepted that inflammation and inflammation associated factors are associated with CAP. Çolak et al^[24]^ found that CAP and PCT could be used as biomarkers in discrimination of CAP and exacerbation of chronic obstructive pulmonary disease. Agnello et al^[25]^ demonstrated that CRP predicted the extent of chest X-ray infiltration and ultimately the severity and PTC levels was correlated with inflammatory factors of CAP patients. Guo et al^[26]^ also found that serial serum CRP3, CRP5, PCT3, PCT5, and PCT5c levels were markedly lower in CAP survivors than deceased patients. In another study, it was reported that higher serum IL-6 and TNF-α levels predicted early death in CAP patients.^[27]^ In the present study, we also found inflammatory factors were elevated in severe CAP patients. Besides, we showed SIRT3 might be associated with the inflammation response in CAP patients, which needs more studies to confirm.

The present study also has some limitations. The size of the study population is small and the underlying mechanism for SRIT3 in CAP is unclear. All these need more studies to further reveal.

## 5 Conclusion

This study demonstrated that lower SIRT3 levels predicted poor prognosis and clinical outcomes of severe CAP patients. SIRT3 was negatively correlated with inflammatory factors in severe CAP. SIRT3 has the potential to be used as a prognosis biomarker in CAP.

## Author contributions

**Data curation:** Wei Zhu, Ping Chen, Li Deng, Liangzi Hu.

**Formal analysis:** Wei Zhu, Ping Chen, Li Deng.

**Investigation:** Wei Zhu.

**Methodology:** Wei Zhu, Ping Chen, Li Deng.

**Project administration:** Wei Zhu.

**Resources:** Wei Zhu, Ping Chen.

**Software:** Wei Zhu, Ping Chen.

**Supervision:** Ping Chen.

**Validation:** Wei Zhu, Li Deng.

**Visualization:** Ping Chen.

**Writing – original draft:** Wei Zhu.

**Writing – review & editing:** Ping Chen, Liangzi Hu, Li Deng.
